# Microwave imaging for human brain stroke detection using frequency domain inverse modelling & phantom experiments

**DOI:** 10.1038/s41598-025-18729-w

**Published:** 2025-10-06

**Authors:** Soumya Prakash Rana, John G. Davis, Kamal Khalil, Michael O’Toole, Stuart Watson, Shijie Liang, Adrian Parry-Jones, David J. Daniels, Anthony J. Peyton

**Affiliations:** 1 Electromagnetic Sensing Group, Department of Electrical and Electronic Engineering, University of Manchester, Manchester, United Kingdom; 2 School of Engineering, University of Greenwich, Medway, United Kingdom; 3 Medical Physics Department, Clinical Engineering, Northern Care Alliance NHS Foundation Trust, Manchester, United Kingdom; 4 Geoffrey Jefferson Brain Research Centre, Manchester Academic Health Science Centre, Northern Care Alliance NHS Foundation Trust, University of Manchester, Manchester, United Kingdom; 5 Division of Cardiovascular Sciences, Faculty of Biology, Medicine and Health, University of Manchester, Manchester, United Kingdom

**Keywords:** Biomedical engineering, Electrical and electronic engineering

## Abstract

This paper reports a study exploring microwave Inverse Synthetic Aperture Radar (ISAR) imaging of biological specimens, with the longer-term goal of assessing its applicability for non-invasive and non-destructive imaging of the human brain in the context of stroke detection and monitoring. The paper describes the design and fabrication of a laboratory testbed developed to examine the feasibility of the ISAR approach. The system includes a custom antenna designed to reduce self-generated clutter and support the imaging process. Water was used as a matching medium due to its specific permittivity-frequency relationship, providing controlled conditions for experimental evaluation. The forward and inverse models were initially tested in simulated environments, and subsequently evaluated using physical measurements on real biological specimens in a bistatic radar configuration, to assess their ability to localize internal anomalies with sub-centimetre resolution across a 26 cm circular imaging area. The reconstructed images from vegetable phantoms such as potatoes and turnips suggest the technique may be capable of detecting internal structural variations. These preliminary findings serve as a foundation for future investigations into human brain imaging applications.

## Introduction

A stroke is a severe medical emergency caused by interrupted blood flow to the brain, often due to a blocked or ruptured blood vessel. This deprivation of oxygen and glucose leads to rapid loss of brain function, with potentially severe consequences including death or long-term disability. Stroke is the second leading cause of global mortality and a major contributor to permanent disability^1^. Strokes can be classified into two main categories: ischemic and hemorrhagic. Ischemic stroke occurs due to diminished blood supply, often caused by thrombosis or embolism. Hemorrhagic stroke results from a ruptured blood vessel in the brain, leading to rapid blood accumulation and tissue compression. Early treatment is crucial for minimizing damage. Ischemic strokes are the most common clinically, and thrombolytic drugs play a key role in treatment by breaking down blood clots. Administering thrombolytics within three hours of symptom onset is time-sensitive^2^. Early diagnosis and vigilant monitoring in the initial hours after a stroke are crucial for recovery and treatment outcomes^3^. Clinically, diagnosis is typically performed using computed tomography (CT) or magnetic resonance imaging (MRI), both of which provide high-resolution imaging of the brain. However, these modalities can be limited by high cost, lack of portability, and delayed accessibility in emergency or bedside settings. Due to their drawbacks, they cannot be conveniently deployed at the patient’s bedside for fast response. Microwave imaging (MWI) has emerged as a potential solution in recent years due to its non-invasive nature, lower cost, and portability^4–6^. Contrasting dielectric properties serve as the basis for MWI. An MWI system utilizes non-ionizing radiation, which is significantly different from ionizing radiation. Ionizing radiation, such as X-rays and gamma rays, has enough energy to remove tightly bound electrons from atoms, potentially causing damage to Deoxyribonucleic acid (DNA) and leading to health risks. In contrast, non-ionizing radiation, like microwaves and radio waves, lacks the energy to ionize atoms or molecules and is considered much safer for biological tissues.

MWI is grounded in studies revealing variations in brain tissue dielectric properties under microwave frequencies, which may assist in identifying conditions such as tissue malignancies, changes in blood supply, and acute ischemia^7,8^. By subjecting head tissues to controlled low levels of microwave energy and collecting the resulting scattered energy through an array of antenna elements, it becomes possible to approximate the dielectric profiles of brain tissues and inform diagnostic assessment. Significant variations in the electric properties of head elements are observed across different microwave frequency bands. For instance, the properties of blood exhibit higher values compared to those of grey and white matter, which may support the detection of hemorrhagic stroke. Conversely, ischemic strokes exhibit tissue properties resembling blood clots, with lower values compared to cerebral matter (e.g., at 1 GHz, relative permittivity is about 36, conductivity about 0.72 S/m) whereas relative permittivity and conductivity of grey matter are 55 and 1.36S/m respectively. These findings have motivated global research into microwave technology for brain imaging and monitoring^9^.

Pioneering research in this field began in 1989 with Lin and Clark^10^, who aimed to detect cerebral edema, excessive water build-up in the brain’s cavities. They used a 2.4 GHz signal and a basic head phantom to simulate a human head, and the collected signals were inverted using back-projection. Since these first reports, researchers have striven to improve each step, recognizing that suboptimal outcomes can hinder efficacy. Some efforts^11,12^ extend resulting images into three-dimensional representations. Additionally, certain studies have incorporated artificial intelligence (AI) techniques, including conventional machine learning and deep learning models, to classify brain conditions (such as healthy or stroke afflicted) even when not prioritising high fidelity image reconstruction, instead relying solely on the capabilities of AI algorithms.

Some studies concentrated on creating lifelike head phantoms mimicking the brain and designing efficient low-power antennas. Mobashsher and Abbosh^13^ utilized MRI and 3D printing to create a human head phantom, validating microwave-based imaging systems. The phantom mimics real tissues across 0.5−4 GHz, enabling repeatable experimentation. Pierre-Henri et al.^14^ explored microwave tomography for stroke imaging using advanced numerical modelling and parallel computing. Their study employed iterative microwave imaging with high-order finite elements and parallel pre-conditioners, aiming for efficient reconstructions using the BRIMG1 system by EMTensor GmbH for stroke detection and treatment monitoring. Rodriguez-Duarte et al.^15^ presented a microwave antenna design for diagnosing and monitoring cerebrovascular conditions. The antenna, embedded in a custom-permittivity block, eliminates the need for liquid coupling media and allows for simple array configurations. Constructed with urethane rubber and graphite powder, it operates within the 800 MHz-1.2 GHz frequency range, meeting device specifications. Experimental validation includes differential signal measurements on a stroke-simulating head phantom. Rodriguez-Duarte et al.^16^ conducted a numerical analysis using a 3D model of a microwave imaging system for monitoring brain stroke progression. The system included 24 printed monopole antennas in custom permittivity dielectric bricks. Numerical simulations evaluated its ability to detect hemorrhages and ischemias, aiding clinical follow-up. The model generated electromagnetic data for an imaging algorithm, offering insights into stroke evolution. Naghavi et al.^17^ introduced an ultra-wideband (UWB) microwave imaging system with an elliptical synthetic aperture radar (ESAR) for human head imaging. Operating in the time domain, it features a miniaturized, low-profile UWB patch antenna. Investigations showed improved range resolution with increased bandwidth. Experimental validation using a 3D MRI derived phantom suggested the system’s potential for early diagnosis and treatment monitoring, including chemotherapy or radiotherapy.

Some research in the literature focuses on image reconstruction algorithms. Rodriguez-Duarte et al.^18^ validated a microwave imaging system for real-time monitoring of post-acute brain stroke. Using a low-complexity sensing device and a multi-frequency imaging algorithm, the system included a novel artefact removal feature. Experiments demonstrated its ability to track hemorrhage and ischemia zones with centimetre-level spatial resolution, offering insights into stroke evolution. Karadima et al.^19^ proposed the DBIM-TwIST algorithm for brain stroke detection, validated using an anatomically accurate head phantom. Simulations in CST Microwave Studio and experimental data processed with DBIM-TwIST showed encouraging results in locating and reconstructing stroke targets. Lei et al.^20^ introduced Adaptive Clustering Distorted Born Iterative Method (AC-DBIM), a modified approach for stroke detection and classification in brain imaging. AC-DBIM clusters reconstructed electrical properties (EPs) during iterations, requiring prior knowledge of bio-tissues. Comparative analysis indicated AC-DBIM’s improved performance in 2D and 3D scenarios, validated through simulations and experimental trials on clinical electromagnetic head scanners. Results highlight its enhanced performance in size and shape reconstruction and reduced errors compared to conventional DBIM approaches. Rui et al.^21^ proposed a novel microwave imaging approach for early stroke diagnosis. Using variational autoencoders, it reconstructs 3D brain electrical properties, improving detail and accelerating computation compared to voxel-based techniques, thereby supporting more accurate diagnosis. Mousavi and Majedi^22^ enhanced microwave imaging for stroke diagnosis, improving the Distorted Born Iterative Method (DBIM) by optimizing the initial guess. Evaluating BIM and DBIM’s dependence on it, the study introduced a three-step process: initial reconstruction using BIM, comparison with MRI-based brain images, and refinement with DBIM. Comparative analysis showed significantly improved accuracy and image quality. Afsari et al.^23^ introduced an optimization strategy for Microwave Tomography (MWT) to address convergence slowness and solution accuracy issues, especially in complex geometries and heterogeneous media. By extending Lagrange multipliers within a Newtonian framework, five steps enhance convergence and accuracy, including parallelizing gradient vectors and optimizing computational efficiency. The approach shows significant improvement in convergence rate and reduction in false-positive or false-negative solutions, seen in brain stroke imaging applications. Anwar et al.^24^ reported a low-complexity wearable RF sensing system for brain stroke and atrophy detection. RF sensors detect cerebral blood density variations and blood clots, employing a customized microwave imaging algorithm for brain reconstruction. Validated through simulations and hardware modelling, it demonstrated effectiveness in detecting blood density variations, stroke targets, and brain atrophy in a realistic brain phantom. Safety is confirmed through Specific Absorption Rate evaluation and thermal conductivity assessment.

### Problem domain & contribution

Researchers face several challenges in microwave brain imaging: (a) microwaves interact in complex ways with brain tissues, resulting in signal distortion that may arise from frequency-dependent changes in dielectric permittivity, unlike air, which typically preserves UWB signal shape; (b) non-linear relationships between incident signals and data complicate reconstruction; (c) limited data collection angles can lead to sparse, inaccurate images; (d) ill-posedness creates multiple potential solutions; (e) noise introduces artefacts that degrade image quality, with thermal noise being one factor. A further important consideration is the external interference from other applications sharing the frequency band, such as global system for mobile (GSM) communications, wireless fidelity (Wi-Fi), and digital television (DTV); (f) complex algorithms and resources are needed for computation; (g) accurate tissue modelling is crucial for precise imaging; (h) hardware design complexities affect data reliability; (i) clinical validation is necessary for accuracy.

In this context, the research reported here is an initial investigation into microwave inverse synthetic aperture radar (ISAR) imaging of biological objects, combined with the use of low ringdown ultrawideband antennas, as the objects of interest are in the range of some millimetres. Antenna ringing has the potential to reduce the range resolution of the system while increasing the self-generated clutter level. The overarching goal is to extend this technology to enable non-invasive and non-destructive imaging of experimental phantoms for stroke detection. Several notable contributions are reported in this paper:

1. Development of a customized antenna with low ringdown characteristics and directional gain, enabling effective suppression of self-generated clutter and suitability for short-range, water-coupled biomedical imaging.
2. Integration of matching media (e.g., water) to ensure adequate electromagnetic coupling in biological environments. Radar sensitivity analysis was conducted using CST simulations to optimize frequency sweep and propagation distance parameters prior to physical measurements.
3. Implementation of a bistatic ISAR formulation adapted to cylindrical geometries and physical rotation. A pseudo-inverse based back-projection algorithm was used to reconstruct spatial scatterer maps, enabling rapid anomaly localization under simplified assumptions of constant RCS.
4. Practical validation of the forward and inverse models using organic biological surrogates (potatoes and turnips) containing artificial dielectric inclusions. While not anatomically or dielectrically equivalent to human tissue, these models allowed initial assessment of resolution and anomaly detectability under realistic scattering conditions.

These contributions represent a prototype-stage microwave imaging system designed for early spatial mapping of dielectric heterogeneities. The results serve as a foundation for future work involving anatomically accurate, frequency-dispersive brain phantoms aimed at stroke detection and diagnostic validation.

The subsequent sections of this article are structured as follows. “Method” elucidates the steps through a flowchart, providing comprehensive details about the proposed forward and inverse model. In “Experimental result analysis”, experimental results are presented, encompassing the design of the antenna, the selection of relative permittivity, outcomes from simulated environments, the intricacies of the experimental setup, and results derived from real-world environments. Finally, “Discussion & conclusion” serves as the conclusion, encapsulating the key findings and paving the way for future research directions.

## Method

**Fig. 1:**
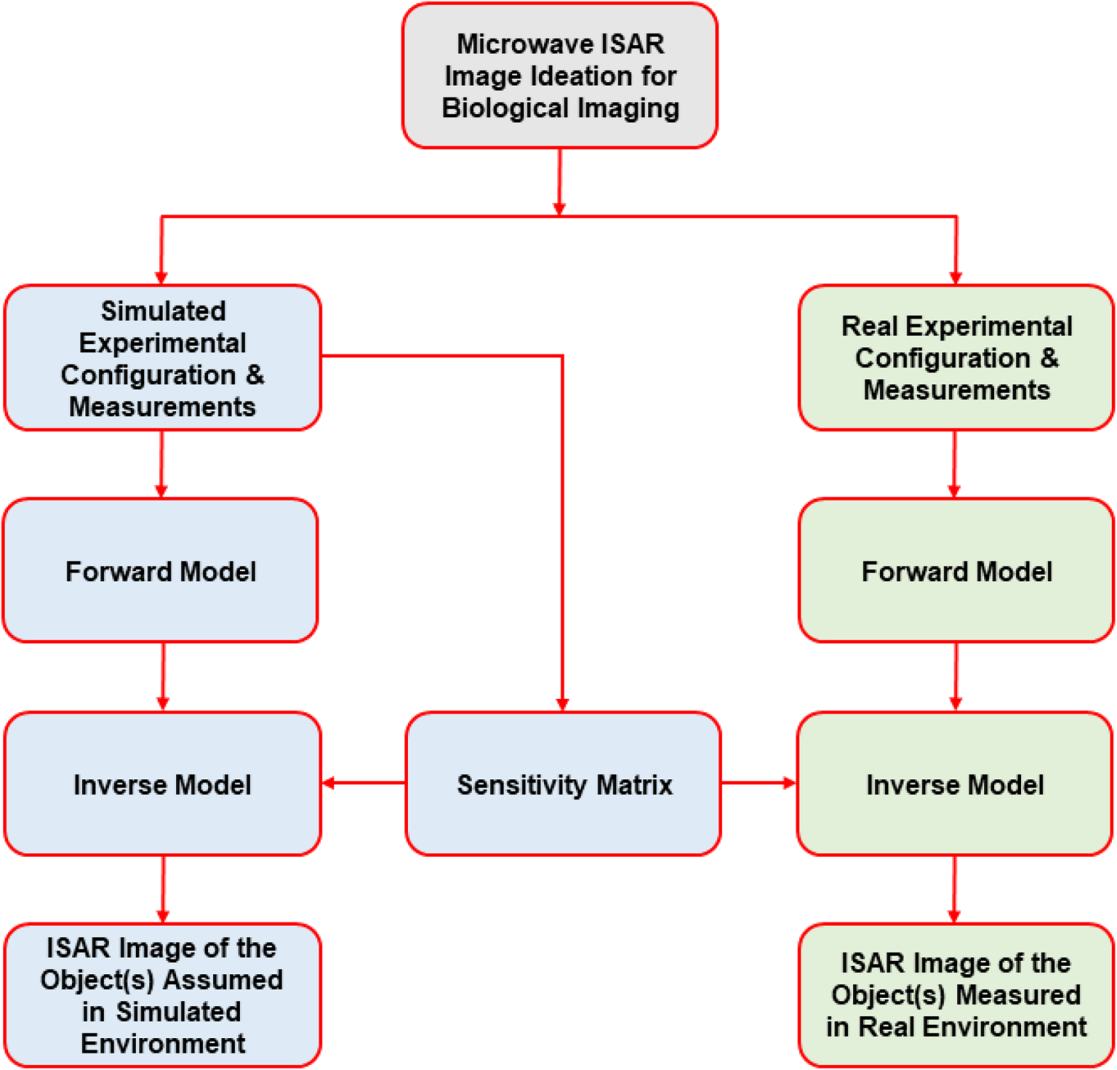
Flowchart illustrating the proposed research methodology.

The proposed research methodology is outlined in Fig. 1, encompassing three main stages: ideation, image reconstruction in a simulated environment, and image reconstruction in a real environment. Initially, the simulated environment was established using a 2D geometric plane, with simulated measurements and sensitivity matrices developed based on antenna theory and scattering properties, as elaborated in “Forward model”. A forward model was constructed for assumed objects, followed by a proposed frequency domain inverse process to generate microwave ISAR images. Once the feasibility was demonstrated with reconstructed images for assumed objects, the same sensitivity matrix from the simulated environment was applied to invert measurements collected for target objects. Operational blocks are represented by three colours: grey for ideation, blue for simulated environment operations, and green for real environment operations.

### Forward model

A bistatic radar configuration has been chosen in polar plane, featuring separated transmitting and receiving antennas, are well-suited for microwave ISAR imaging. These configurations offer advantages such as reduced clutter, providing clearer received signals, and flexibility in antenna placement for optimal imaging geometry.

**Fig. 2:**
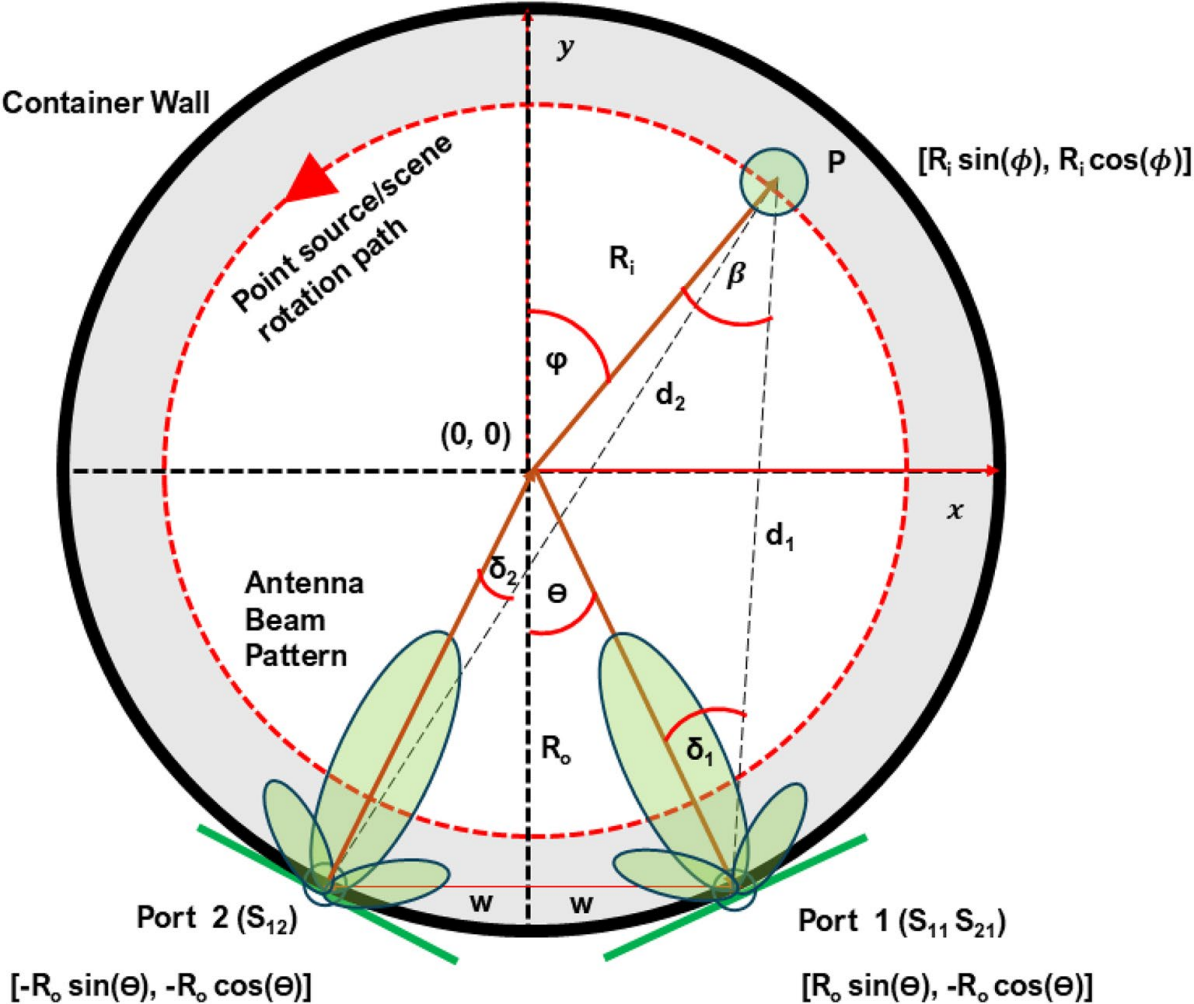
Illustration depicting the propagation geometry, including the positions of $T_x-R_x$ antennas, measurement space, and objects, in both simulated and real environments, crucial for developing the theory to calculate scattering parameters. Here, $\delta _1$ and $\delta _2$ indicate the angles between the antenna boresights (transmit and receive) and the direction to the target *P*, not relative to the global coordinate axes.

The geometry of microwave system is illustrated in Fig. 2. Microwave measurements were conducted within a polar plane, encompassing the inside of a container filled with a matching liquid. The polar plane’s centre was designated as (0, 0), and the container, had a radius of $R_0$. Transmitting $(T_x)$ and receiving $(R_x)$ antennas were positioned just outside the container, each with a radius of $R_0$ and creating a $\theta$ angle with the y-axis. The coordinates of the $T_x-R_x$ antennas in the polar plane were $(R_0 Sin \theta , R_0 Cos \theta )$ and $(-R_0 Sin \theta , -R_0 Cos \theta )$. The directional beam pattern of these antennas was represented in green. The two antennas were assumed to be separated by a distance of 2*w*. For illustration, a point source object with an RCS of 1$m^2$ was assumed at point *P*, marked with a green circle, and its polar coordinates were $(R_i Sin \phi , R_i Cos \phi )$, creating a $\phi$ angle with the y-axis. Point *P* represents a generic scatterer location in the scene, used to illustrate the geometric relationships in the bistatic configuration. It can be placed or suspended anywhere within the tank volume and does not touch the container wall during experimental measurements, ensuring a more accurate interpretation of the scattering effect.

If the angular span or coverage of the $T_x-R_x$ pattern in a specific direction is denoted as $\delta _2$ and $\delta _1$, respectively, then mathematically $\delta _2$ and $\delta _1$ can be expressed and shown in Eq. 1 and 2. In this configuration, $\delta _1$ and $\delta _2$ denote the angles between the main beam direction of the transmitting and receiving antennas, respectively, and the vector connecting each antenna to the target at point *P*. These are not referenced to the vertical (*y*) axis but are local to each antenna’s orientation.

1 $$\begin{aligned} \delta _1 = tan^{-1} \frac{R_i sin(\theta + \phi )}{R_0 + R_i cos(\theta - \phi ) } \end{aligned}$$

2 $$\begin{aligned} \delta _2 = tan^{-1} \frac{R_i sin(\theta - \phi )}{R_0 + R_i cos(\theta - \phi ) } \end{aligned}$$

Within that antenna coverage, the distances between the object (*P*) and $T_x$ $(d_2)$ and between the object (*P*) and $R_x$ $(d_1)$ were denoted accordingly. The distances $d_1$ and $d_2$ can be expressed mathematically and shown in Eq. 3 and 4,

3 $$\begin{aligned} d_1 = \sqrt{(R_i Sin \phi - R_0 Sin \theta )^2 + (R_i Cos \phi + R_0 Cos \theta )^2} \end{aligned}$$

4 $$\begin{aligned} d_2 = \sqrt{(R_i Sin \phi + R_0 Sin \theta )^2 + (R_i Cos \phi + R_0 Cos \theta )^2} \end{aligned}$$

In the context of a bistatic radar system, the received power is intricately linked to crucial parameters such as the effective area of the receiving antenna, wavelength, bistatic range, and transmitted power. Consequently, understanding the antenna’s capability to capture incoming signals is essential. The expression for the effective aperture of the receiving antenna, denoted as $(A_0)$, is mathematically defined in Eq. (5). In this equation, $Z_0$, $P_t$, and $\lambda$ represent the impedance of free space, transmitted power, and the wavelength of the transmitted signal, respectively. This mathematical relationship provides insights into the antenna’s performance and its role in bistatic radar scenarios.

5 $$\begin{aligned} A_0=\sqrt{\frac{Z_0P_t}{4\pi }} .\frac{\lambda }{4\pi } \end{aligned}$$

Consequently, the bistatic radar scattering equation, particularly the received signal $(S_r)$ concerning pertinent parameters, can be articulated as follows, shown in Eq. (6),

6 $$\begin{aligned} S_r\left( \omega ,\phi \right) \ ={\ \ A_0\left( \frac{\sqrt{G\left( \delta _1\right) G\left( \delta _2\right) \sigma \left( \beta \ \right) }}{d_1{\left( \phi \right) d}_2\left( \phi \right) }\right) } e^{j\left( \frac{\omega \sqrt{\varepsilon _r}}{c_0}\right) \left[ d_1\left( \phi \right) \ \ +\ \ d_2\left( \phi \right) \right] } \end{aligned}$$

where, $S_r(\omega , \phi )$ is the received signal with $\omega$ angular frequency at a specific angle $\phi$, $A_0$ is the effective aperture of the receiving antenna, $G(\delta _1)$ and $G(\delta _2)$ represent the antenna gains in specific directions or subtended angles (shown in Eqs. 1 and 2), $\sigma (\beta )$ denotes the radar cross-section of the target at the aspect angle $\beta$, $d_1(\phi )$ and $d_2(\phi )$ are the distances from the target to the receiving and transmitting antennas respectively at the angle $\phi$, $\varepsilon$ is the relative permittivity and square root of the relative permittivity $\sqrt{\varepsilon }$ is the relative permittivity in a medium, $c_0$ is the speed of light. This equation expresses the received signal in a bistatic radar scenario, considering the geometry, antenna characteristics, and target properties. The parameters related to gains, distances, radar cross-section, and electromagnetic properties contribute to the overall received signal. The exponential term with $\sqrt{\varepsilon }$ in the complex exponential represents the phase shift due to the medium’s relative permittivity. Therefore, the scattering parameter $S_r(\omega , \phi )$ represents the $S_{21}$, is the forward voltage gain describes the electrical behaviour of linear electrical networks when undergoing various steady state stimuli by electrical signals (i.e., behaviour of linear electrical networks when microwave is transmitted through a medium to measure the scenario).

Consequently, iterating the $S_{21}$ parameter across a designated frequency range at various azimuth angles across the polar plane yields the forward voltage gains within the measurement scenario, encompassing the matching liquid or medium, from distinct viewing angles in the horizontal plane. Altogether, the $S_{21}$ parameter across the polar plane provides the forward model of the microwave experiment, which is rearranged and presented in Eq. (7) following the Eq. (6) with the variation of frequency and $T_x-R_x$ positions in azimuth angle. The variables *m* and *n* represent change of angular frequency $\omega$ (where $m=1,\ldots ,M$) and change of azimuth angle $\phi$ (where $n=1,...,N$). In this synthetic aperture configuration, $\phi$ represents the angular position of a target voxel in the scene with respect to the origin. During measurements, the scene (including the target) is physically rotated relative to the fixed antenna pair. Thus, $\phi$ also indexes the scene’s angular rotation step, which modulates the path lengths $d_1$ and $d_2$ as functions of $\phi$ in the forward and inverse models. This dual role of $\phi$ enables the reconstruction of target locations across the full angular aperture.

7 $$\begin{aligned} S_r(\omega _m, \phi _n)= A_0\omega _m \sum _{m=1}^{M} \sum _{n=1}^{N} \left( \frac{\sqrt{G(\delta _1(\omega _m, \phi _n)) G(\delta _2(\omega _m, \phi _n)) \sigma }}{d_1(\phi _n) d_2(\phi _n)} \right) e^{j (\frac{\omega \sqrt{\varepsilon _r}}{c_0}) [d_1(\phi _n) + d_2(\phi _n)]} \end{aligned}$$

### Sensitivity matrix

The sensitivity matrix captures the interplay between alterations in the forward voltage gain $S_{21}$ and changes in the properties of the imaged object. Essentially, it encapsulates the dielectric parameters (i.e., permittivity and conductivity) of the imaging space within a matching medium and is established during the forward modelling phase. Consider an imaging space in the polar plane with dimensions $(p \times q)$. Following Eq. 7, the scattering parameters $S_r(\omega _m, \phi _n)$ are computed for all pixels within the imaging or object space. Organizing these parameters into a matrix yields the sensitivity matrix of the proposed ISAR imaging system, as depicted in Eq. 8. Here, $S_r(\omega _m, \phi _n)$ is arranged with varying angular frequencies $\omega$ ($m=1,...,M$) and azimuth angles $\phi$ ($n=1,...,N$) for each pixel, with columns corresponding to individual pixels. The subscript *r* denotes the pixel number, with a total of $(p \times q)$ pixels considered, resulting in $(p \times q)$ columns in the matrix.

8 $$\begin{aligned} J= \begin{bmatrix} S_{r_1}^{\phi _1}(\omega _1) & S_{r_2}^{\phi _1}(\omega _1) & . & . & . & S_{r_(p\times q)}^{\phi _1}(\omega _1)\\ . & . & . & . & . & .\\ . & . & . & . & . & .\\ . & . & . & . & . & .\\ . & . & . & . & . & .\\ . & . & . & . & . & .\\ S_{r_1}^{\phi _n}(\omega _m) & S_{r_2}^{\phi _n}(\omega _m) & . & . & . & S_{r_(p\times q)}^{\phi _n}(\omega _m)\\ \end{bmatrix} \end{aligned}$$

### Proposed inverse model

In realistic electromagnetic modeling, the dielectric properties of an object are frequency-dependent and exhibit dispersion across angular frequencies ($\omega$). Consequently, both the scattering parameters $S(\omega , \phi )$ and the medium’s electromagnetic response vary with frequency and azimuthal angle ($\phi$), particularly in biological tissues. These parameters encapsulate the electromagnetic responses of the object within a defined geometric space. Each point or pixel (*P*) within this object space exhibits unique dielectric characteristics across different frequencies and azimuth angles, forming a response matrix (*J*), representing the relationship between changes in the forward voltage gain *S* (practically i.e., $S_{21}$) and changes in the properties of the imaged object. Assuming a constant RCS value of 1 for each pixel *P*, these individual responses are organised into a matrix representation. The electromagnetic interactions between the dielectric properties of the geometric points *P*, distribution of the desired dielectric responses represented by matrix *J*, and the measured scattering parameters *S* enable the formulation of a linear equation system in matrix form,

9 $$\begin{aligned} \begin{aligned} S&= J.P \\ \Rightarrow P&= S.J^{-1} \end{aligned} \end{aligned}$$

This matrix equation is proposed and subsequently solved to determine the spatial distribution of effective backscatter strength *P*, which corresponds to localized RCS values rather than intrinsic dielectric parameters. These variables are then utilized to reconstruct ISAR image(s) of the objects under study. The size of *J* is $( \omega \times \phi ) \times (p \times q)$, the significance of $\omega$, $\phi$, *p*, *q* have been stated above. Practically, *J* is not a square matrix or singular matrix and the inverse does not exist. Therefore, the generalization of the inverse of a matrix, pseudo-inverse or Moore-Penrose pseudo-inverse^25,26^ has been performed to solve the Eq. 9 and allowed to find the “best fit” solution even when *J* is not invertible. As $( \omega \times \phi ) \ge (p \times q)$, then $J^TJ$ is invertible and $J^{-1}=(J^TJ)^{-1}J^T$ and so $J^{-1}.J=I$ i.e., $J^{-1}$ is the left inverse of *J*. Therefore, the Eq. 9 has been solved in Eq. 10 determining the dielectric properties of imaged object based on the measured scattering parameters and the sensitivity matrix.,

10 $$\begin{aligned} P = S.(J^TJ)^{-1}J^T \end{aligned}$$

The pseudoinverse of $J^{-1}$ has been computed in the MATLAB environment using singular value decomposition (SVD) to obtain the solution, where the estimation treats singular values less than a tolerance as zero. The proposed work has estimated the sensitivity matrix *J* considering a polar space of $(64\times 64)$ with 8 cm and 10 cm radius on the cartesian plane resulting in the reconstructed image P as a $(64\times 64)$ dimensional image. This image has been linearly interpolated to $(2401\times 2401)$ for creating the final presentation version of the ISAR image of the object(s). The dimension $(64\times 64)$ was chosen experimentally to ensure the process runs fast (practically runs in less than 60 seconds) on a DELL Precision 5820 workstation features an Intel Xeon W-2275 CPU runs at a base frequency of 3.30 GHz, with a turbo boost up to 3.31 GHz, 128 GB RAM, running MATLAB 2023b in a 64-bit Windows 10 professional environment.

## Experimental result analysis

This section describes the outcomes from both simulated and experimental measurements, structured across three stages. The computations for the results of the forward and inverse models adhere to the theoretical framework expounded in “Forward model” and are detailed in “Results obtained from simulated forward & inverse model”. Subsequently, “Real experimental set-up” provides an exposition on the actual experimental setup employed. Finally, “Results obtained from measured forward & inverse model” presents the results achieved from the forward and inverse models using the measured data. The subsequent subsections offer a detailed breakdown of each stage.

### Designed antenna & selection of relative permittivity

**Fig. 3:**
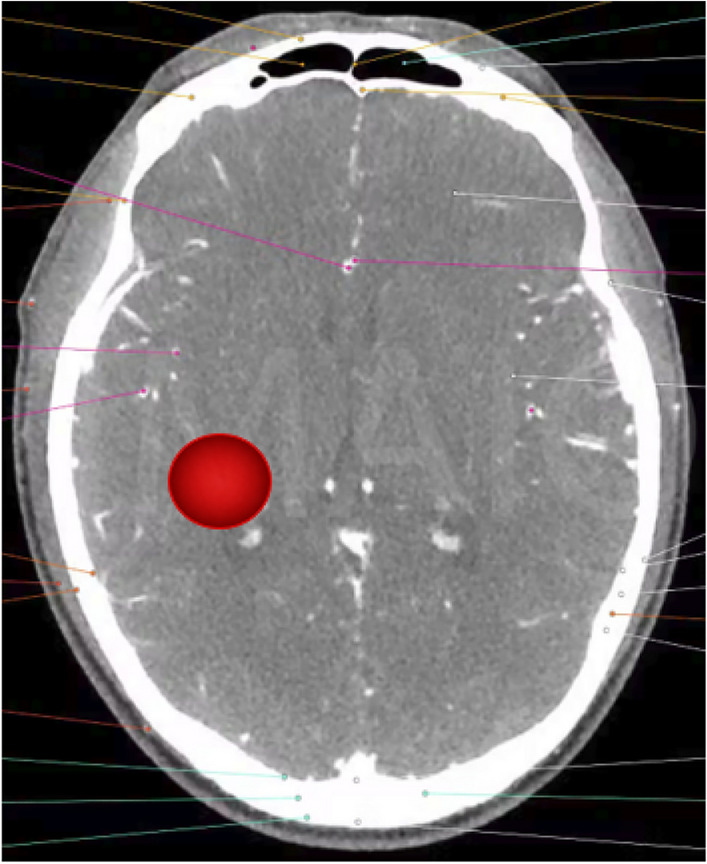
Simulated location of an assumed stroke positioned 5 cm beneath the scalp surface in a human head model.

The synergy between antenna performance and the choice of permittivity is essential for achieving accurate and meaningful microwave imaging for biological elements. Both factors are crucial to optimize the imaging system’s sensitivity, resolution, and capability to reveal important details about the dielectric properties of the imaged specimens. Therefore, there are two aspects considered before running the real measurement antenna performance and determination of the permittivity. In order to establish the performance needed from the antenna an estimate of the signal levels that might be expected is useful. Based on data published by Gabriel^27–30^ the levels of attenuation, transmission losses and reflection losses at the various interfaces can be estimated assuming a water-based coupling fluid between the antenna and the skin. A first order estimation of the overall losses at 1 GHz that might be expected from the site of a stroke 5 cm the surface of the head and shown in Fig. 3 are as follows on the assumption of discrete boundaries.

From Table 1 and 2 it can be seen that the measuring system and antenna whether a single element or a separated pair, one transmitting and one receiving, needs to have a time domain response that allows an adequate signal to clutter performance at a range of 5 cm given that the return signal is in the region of -65 dB to - 75 dB. At 1 GHz the relative dielectric constant of grey matter is in the order the 50 hence the residual clutter needs to less than -70 dB several nanoseconds after transmission. This is a challenge for UWB antenna design. However, the use of element resistive loading, antenna shaping and Radar Absorbing Material (RAM) screening of both the transmit and receive antenna.

**Table 1:** Dielectric properties of various biological tissues at 1 GHz including conductivity, permittivity, and wavelength

	Frequency (Hz)	Conductivity (S/m)	Relative Permittivity	Loss Tangent	Wavelength (m)
Blood	$1 \times 10^{9}$	1.580	61.070	0.466	0.0374
Bone cortical	$1 \times 10^{9}$	0.160	12.360	0.226	0.0847
Cerebro spinal fluid	$1 \times 10^{9}$	2.460	68.440	0.645	0.0346
Bone cortical	$1 \times 10^{9}$	0.160	12.360	0.226	0.0847
Brain grey matter	$1 \times 10^{9}$	0.990	52.280	0.339	0.0409
Brain white matter	$1 \times 10^{9}$	0.620	38.580	0.290	0.0478

**Table 2:** Estimated signal attenuation and loss contributions at 1 GHz from a target located 5 cm beneath the head surface.

Parameter	Value at 1 GHz	Value in dB
Loss in skull bone 1 cm thickness	1 cm $\times$ 2 $\times$ 1 dB cm$^{-1}$	-2.0
Loss in grey matter 3 cm path	3 cm $\times$ 2 $\times$ 2.1 dB cm$^{-1}$	-12.6
Return loss grey matter to blood	-	-13.0
Additional transmission coupling losses*	-	-2.0
RCS of blood section (3 cm diameter sphere)	-	-30.0
Spreading loss at 5 cm	-	-8.0
Total losses	-	-67.6

**Fig. 4:**
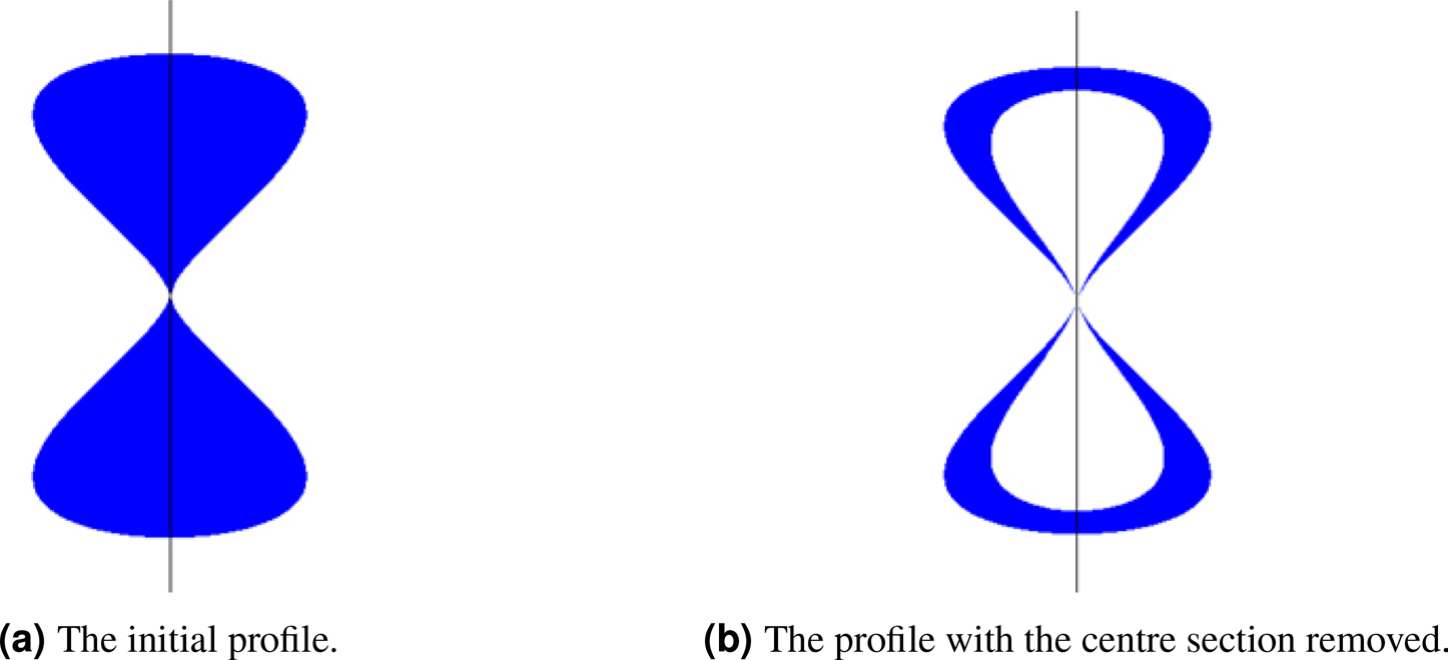
Geometry of the planar ACD antenna.

**Fig. 5:**
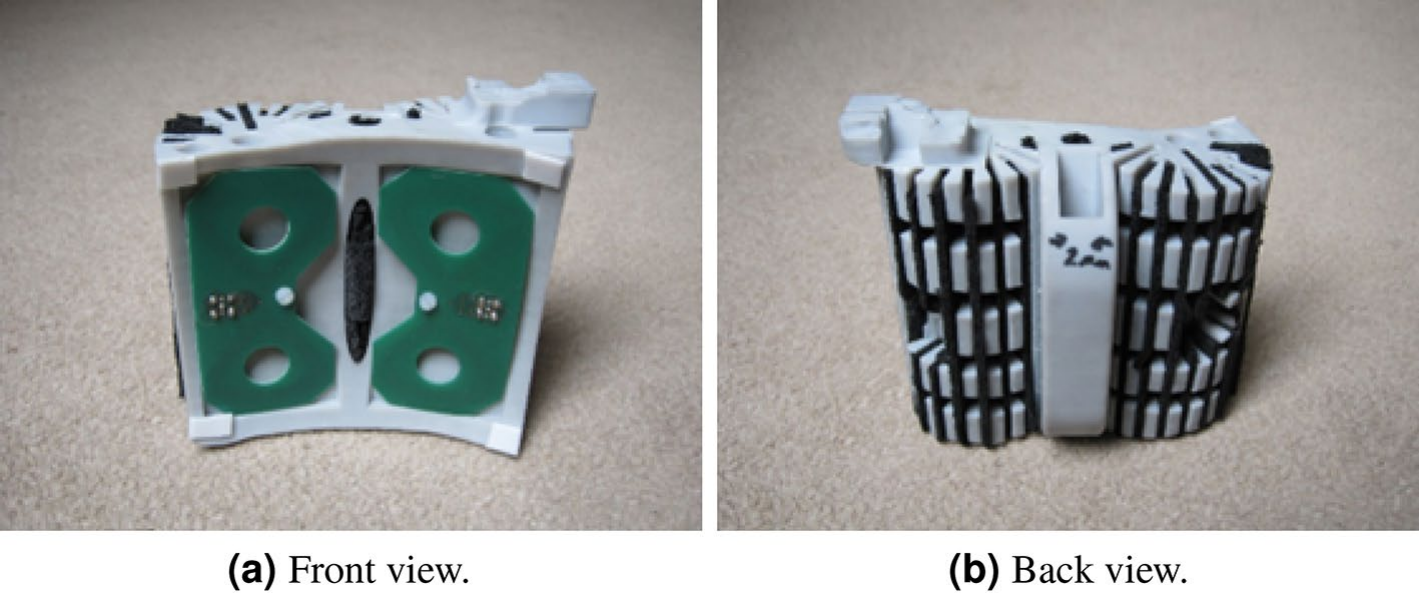
Physical realisation of planar ACD antenna.

To achieve compact radiated wave packets, antennas require wideband performance with a minimum frequency/phase response. The biconical antenna has served as the basis for many UWB antenna designs. The infinite biconical antenna only radiates from its feed point and hence exhibits ultra-wideband performance, if however, it is shortened, then the boundary between the antenna and free space at the ends is the source of charge acceleration and hence radiation and this has the effect of imposing a limitation on not only on bandwidth, but its impulse response. The asymptotic conical dipole (ACD) led to its widespread use in measuring electromagnetic pulse fields where its time domain properties were advantageously exploited. This medical application only permitted a planar version and minimising charge acceleration required that sudden changes in profile and antenna thickness should be avoided. This leads to the profile as shown in Fig. 4a. Analysis of the current profiles on the solid version showed the majority of the current flows on the edge of the antenna hence the centre section may be removed as in 4b. This then serves as the basis for a loading profile along the length of the elements following a well-known Wu-King profile. The final realisation of the antenna together with its RAM screening is shown on Fig. 5a, b, and the length of the antenna is approximately 80 mm.

**Fig. 6:**
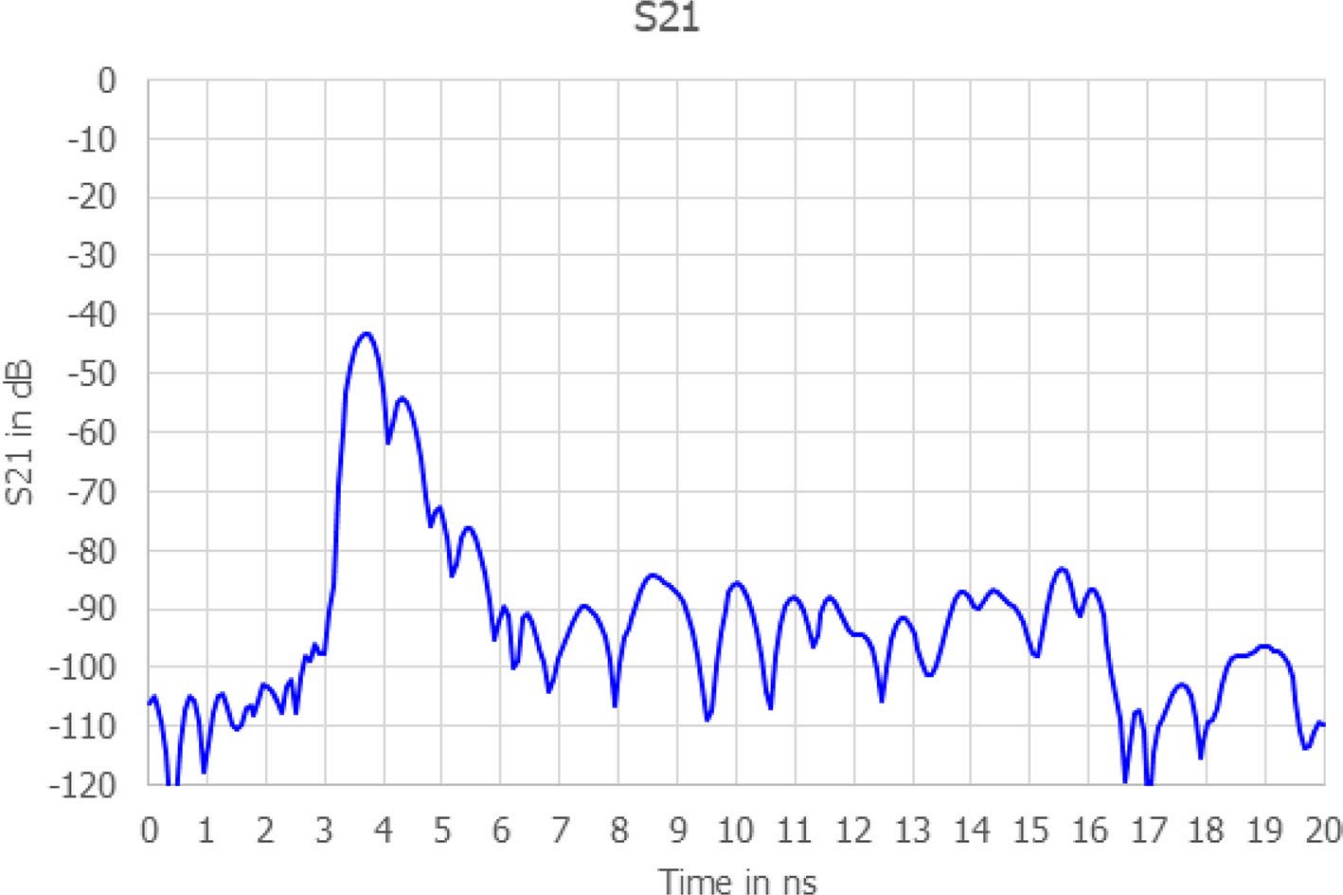
Measured transmission coefficient ($S_{21}$) between a pair of planar ACD antennas in free space, demonstrating low ringdown and rapid signal decay.

The key performance metric for the antenna is a measurement of $S_{21}$ in free space and this is shown in Fig. 6. The key points revealed by this measurement are that the initial cross coupling which occurs at 3.8 ns is – 43 dB and decays to -90 dB at 6 ns. At 5.8 ns s21 is -89 dB which is some 20 dB less than the expected signal at that equivalent time. Therefore, the antenna is a suitable design for this application and was used to gather the results described in this paper.

**Fig. 7:**
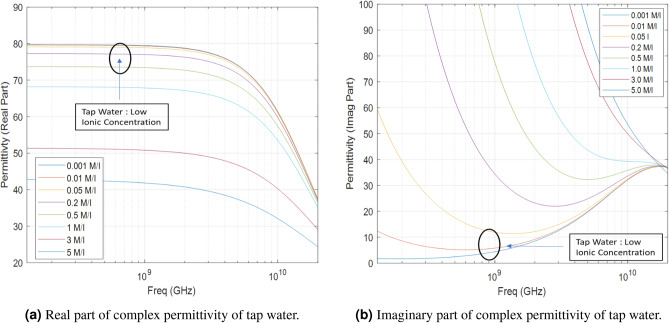
Application of Cole-Cole^31^ model to estimate the complex permittivity of tap water.

As water has been used as matching liquid in this work which mimics the dielectric properties of some biological tissues. The popular Cole-Cole model^31^ has been studied to describe the frequency-dependent behaviour of dielectric properties of water, the relationship between permittivity-frequency, shown in Fig. 7. Figure 7 displays the outcomes of Cole–Cole model computations, depicted as a frequency-dependent function, for water solutions demonstrating diverse salinity levels (ionic concentration). The charts illustrate that the estimates of complex permittivity obtained from the pulse delay analysis of the water, characterized by a low ionic concentration (approximately $80 - j5$). This correlation broadly affirms the soundness of the usage of water in microwave ISAR imaging in this stage. While CST and water coupling are widely used individually, our integration of full wave simulation informed forward modelling, biological phantom experiments, and real time back-projection creates a practical and reproducible ISAR pipeline for brain anomaly detection.

Further, the experiments have been performed in CST simulation environment to understand the interaction between a strong reflector (a 7 mm diameter metal rod) within two different medium (air and water) and radar signal strength over changing the rod’s distance (moving towards and away from radar). The antenna was fully modelled in CST Studio Suite, including dielectric substrate, ground plane, SMA port, and elliptical taper. Simulations provided complete far field radiation patterns and mutual coupling parameters for use in theoretical modelling. There are two environments that have been setup in CST simulation software with empty (air) and water inside tank of 20 cm diameter, a 7 mm diameter metal rod, bistatic radar operating at 0 GHz to 6 GHz. The rod has been moved linearly from -10 cm (other end of the tank or furthest point from radar within tank) to 10 cm (nearest point from radar within tank). The frequency responses have been recorded to understand the received signal strength over distance.

**Fig. 8:**
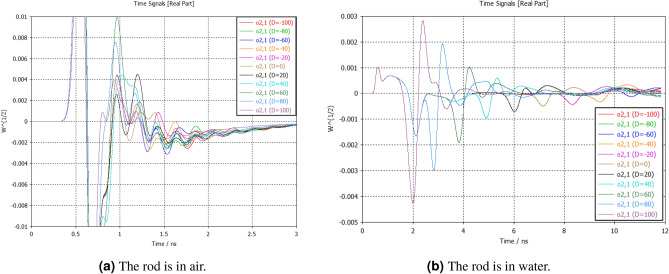
Radar’s sensitivity in air and water medium with a 7 mm metal rod in CST simulation environment.

Figure 8a, b show the change of received power varying metal rod’s distance in air and water in CST simulation environment where *x* and *y* axis represent the propagation delay and received power respectively. Shorter delays indicate closer proximity of the rod to the radar, while longer delays suggest greater distance in both mediums. As the rod moves from -10 cm (farthest from the radar) to 10 cm (nearest to the radar), the received power responses change as a function of range. This change indicates alterations in the echo signal strength received by the radar caused by the presence and position of the rod. As the rod moves closer to the radar (from -10 cm to 10 cm), the frequency responses exhibit consistent trends in received power. However, air and water have different permittivity of approximately 1 and 80 respectively which make difference in received power, propagation speed, signal attenuation, reflection, resolution, and interference.

Differentiation of reflections in air are more challenging compared to water due to their different permittivity. In water, the higher permittivity leads to stronger and more easily identifiable reflections, which can be accurately reconstructed to extract information about submerged objects. Additionally, the slower propagation speed of electromagnetic waves in water allows for longer integration times and more precise measurements, further aiding in the identification and reconstruction of reflections. In contrast, air has a lower permittivity, resulting in weaker reflections that can be more easily attenuated and obscured by noise or interference. This can make it more difficult to differentiate between reflections from different objects or surfaces in the air, especially in environments with high levels of clutter or variability. Overall, the higher permittivity and slower propagation speed of water make it generally more favourable for accurate reflection identification and information reconstruction compared to air.

### Results obtained from simulated forward & inverse model

**Fig. 9:**
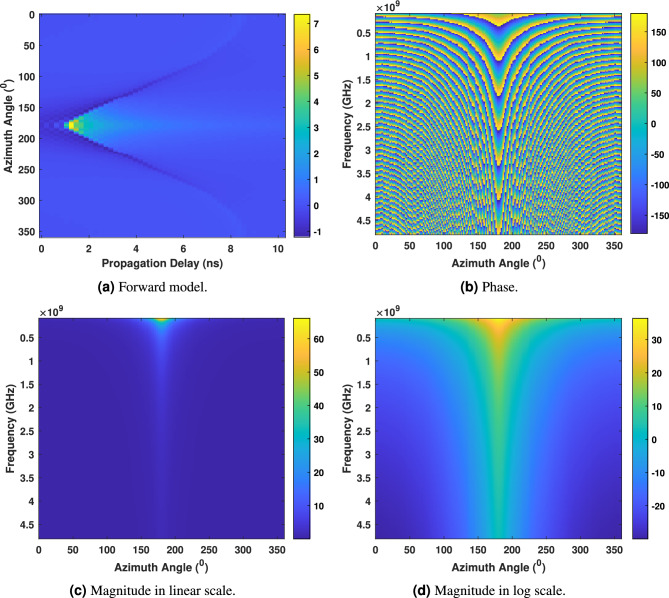
Forward model, phase, and magnitude when single object positioned at (0, -6 i.e., 6 cm from centre at $180^\circ$) is assumed in an simulated environment.

$S_{21}$ measurements have been simulated for a point source object assumed at ${180}^\circ$. These measurements, representing the ratio of output power to input power in a two-port network, focus on a target object, the radar’s point source. The dielectric properties of the target, including relative permittivity and conductivity have been generated to observe the microwave signal response of an object perfectly conducting sphere of projected cross-sectional area that will have an RCS of 1 ${\rm m}^2$. A uniform radar cross-section (RCS) of 1 ${\rm m}^2$ was assumed across all voxels in the inverse model. This simplification facilitates matrix inversion and model benchmarking but does not reflect the realistic variation in scattering strength observed in biological tissues. In practice, RCS varies significantly with frequency, tissue composition, water content, and structural heterogeneity. This limitation may lead to overestimation or underestimation of scattering intensity, thereby affecting tissue contrast in the reconstructed images of real biological elements. Therefore, in the “Real experimental set-up” and “Results obtained from measured forward & inverse model” simple biological elements were tested under this assumption to assess the system’s feasibility and inform future directions. The simulated measurements cover a frequency range from 0.1 GHz to 4.8 GHz, providing a detailed analysis of the target’s characteristics. This range is divided into 200 bins, enabling a thorough examination of the target’s frequency response at different intervals. The simulation has been executed in a polar plane with a diameter of 17 cm (same as the used in real measurement), combined with 360 transmitting-receiving antenna positions. This configuration ensures a comprehensive ${360}^\circ$ view of the target during the simulation. The $S_{21}$ simulated measurements are then transformed using IFFT observe if the time domain signals appropriately represent the target scene, shown in Fig. 9a. The *x*-axis represents the propagation delay (in nanoseconds), and the *y*-axis represents the azimuth angle (in degrees). The colour scale on the right-side ranges from -1 to 7, indicating the intensity of the radar return at each point and the signogram at ${180}^\circ$ shows higher values indicate the presence of the target. Figure 9b–d display the phase shift, magnitude in linear and logarithmic scale respectively of the signal at different frequencies due to the presence (i.e., assumption) of the object, where the *x*-axis represents the azimuth angle in degrees, and the y-axis represents the frequency in GHz.

**Fig. 10:**
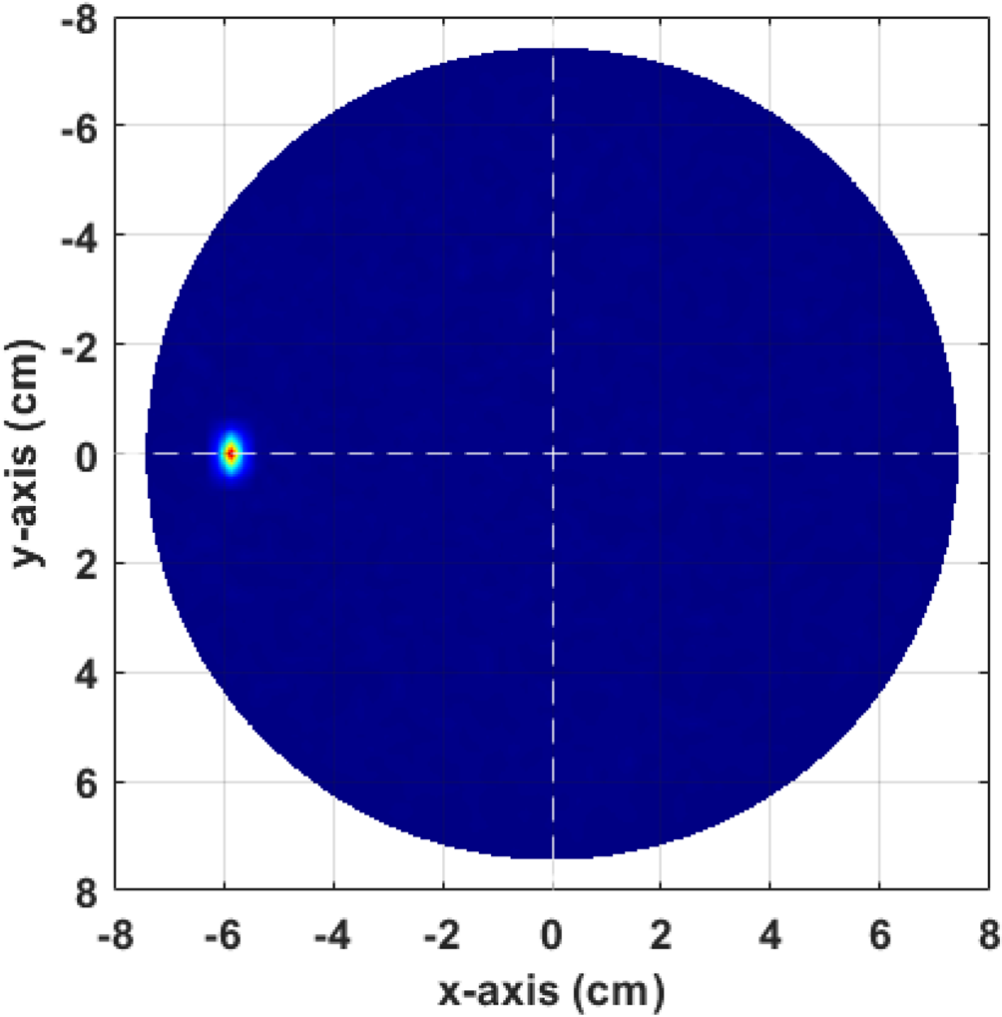
Reconstructed microwave ISAR image when a single object assumed in the simulated environment.

The inverse model has been solved following the linear inverse model stated in “Proposed inverse model” and back-projected in the polar plane to create the microwave image of that assumed target at (0, -6) or 6 cm from centre at $180^\circ$. The reconstructed image is shown in Fig. 10, with the single point like target correctly positioned and well resolved.

**Fig. 11:**
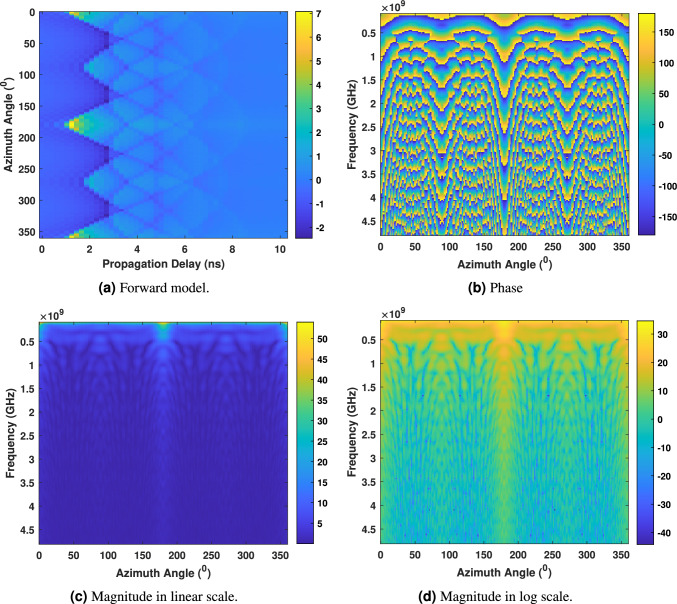
Forward model, phase, and magnitude when multiple objects assumed in a simulated environment.

In the second simulated scenario, we investigate the variation in dielectric properties and observe the responses of the forward and inverse models when multiple objects are present at different distances from the radar across various quadrants of the polar plane. For this scenario, $S_{21}$ measurements are generated for nine-point source objects distributed across the polar plane. This includes four objects on the positive and negative axis lines of *x* and *y* (i.e., ${180}^\circ$ of each axis), four objects at approximately a ${45}^\circ$ angle within each quadrant, and one object at the center. The experimental setup, frequency range, number of bins, and transmitting-receiving antenna positions remain consistent with the parameters used previously. The dielectric properties generated for each frequency bin, represented as $S_{21}$ measurements, have undergone conversion to time domain responses through IFFT. The results, depicted in Fig. 11a, have been examined to observe variations in amplitudes and accurately represent objects at different distances from the radar positions within the forward model. Figure 11b depicts the phase shift in relation to the azimuth angle (on the *x*-axis) and frequency (on the *y*-axis). Notably, the signal’s phase (in degrees) undergoes variations concerning frequency. Substantial changes are observed when the assumed objects are positioned proximate to the antennas, specifically two objects along the positive and negative *x*-axis lines at $0^\circ$ and ${180}^\circ$ of the polar plane. The phase shift diminishes when the objects are slightly more distant, positioned along the positive and negative *y*-axis lines at ${90}^\circ$ and ${270}^\circ$. Furthermore, reduced phase shift is evident for objects assumed at ${45}^\circ$, ${135}^\circ$, ${225}^\circ$, and ${315}^\circ$, placed closer to the centre than the previous two placements. The least phase shift is observed for the object assumed at the centre. These phase alterations align realistically with the expected scattering properties based on the electromagnetic characteristics and geometry of the objects. Examining the relationship between frequency and magnitude, as illustrated in Fig. 11c, d with azimuth angle on the *x*-axis and frequency on the *y*-axis, reveals heightened magnitudes corresponding to locations with increased phase shifts.

**Fig. 12:**
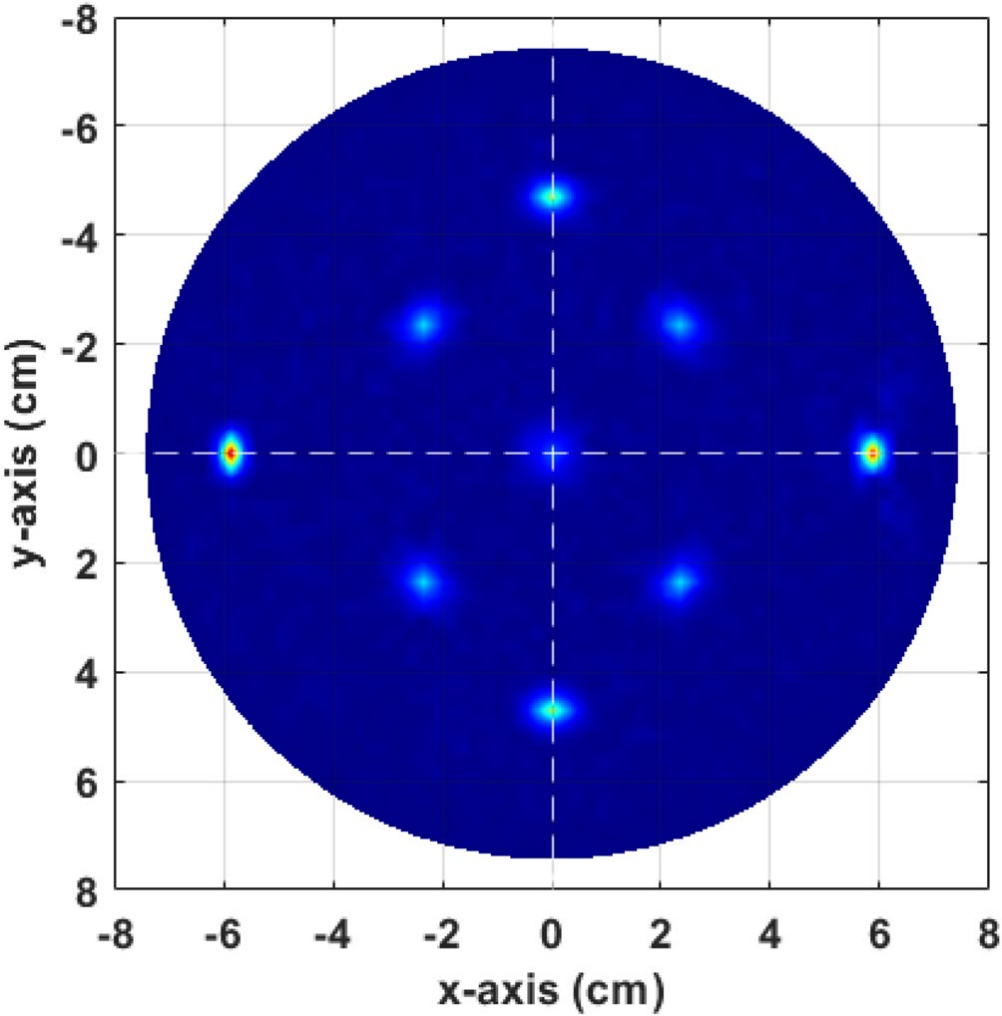
Reconstructed microwave ISAR image when a multiple objects assumed in the simulated environment.

After solving the forward model and observing the outcomes, the inverse model was employed, utilising the linear inverse model described in “Proposed inverse model”. This process involved back projecting the linear magnitude in the polar plane to generate a microwave image of the nine presumed objects distributed across the polar plane at varying distances from the radar and centre. The resulting reconstructed image, shown in Fig. 12, encapsulates the quantitative properties derived from the forward model, including amplitude in the time domain, phase, and magnitude. The image provides a realistic depiction of the observed objects in a ${360}^\circ$ microwave spotlight view.

Upon successful validation of the proposed forward and inverse models with simulated data, an experimental setup was implemented to measure the dielectric properties of real objects. However, a significant challenge arises in the assumption of equal reflectivity in all directions (i.e., RCS of 1 m²) in the inverse model, which is a common hurdle faced by numerous methods in the field of microwave ISAR imaging. The present work aims to address and optimize these challenges, with early outcomes showing promise.

### Real experimental set-up

**Fig. 13:**
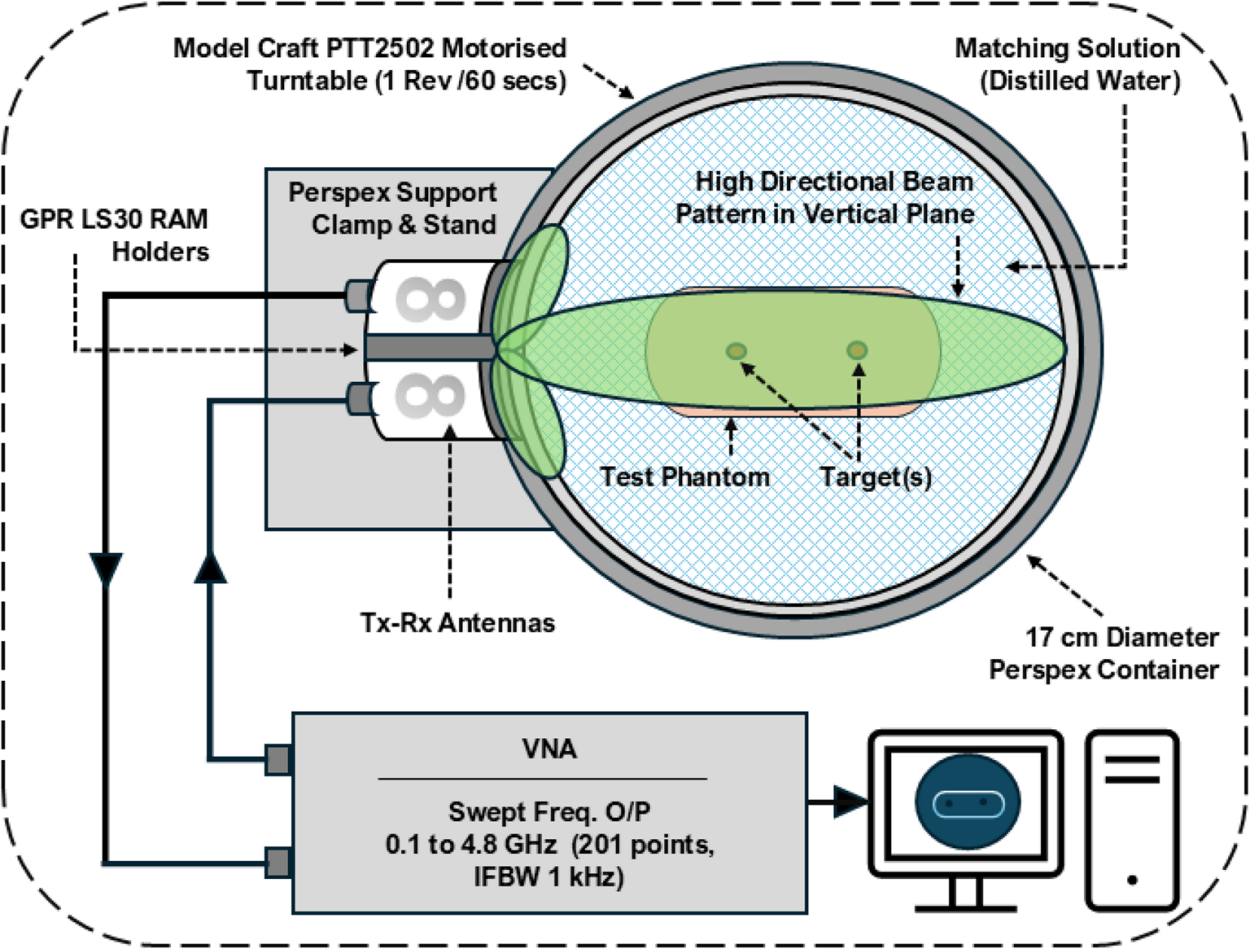
Experimental setup diagram for measuring real biological objects and testing the proposed microwave ISAR image reconstruction model.

The experimental setup (shown in Fig. 13) includes mounting the transmitting-receiving antennas securely on an adjustable support system using the Perspex support clamp and stand. The model craft PTT2502 motorized turntable controls azimuthal rotation, with a rotation rate of 1 revolution per 60 seconds, enabling precise directional measurements. Antennas are configured to maintain a high directional beam pattern in the vertical plane, and a Copper Mountain VNA, set for a swept frequency output of 0.1 to 4.8 GHz (200 points, IFBW 1 kHz), is connected to the antennas for transmission and reception measurements. A short-open-load-through (SOLT) calibration was performed before each measurement session to remove systematic errors and establish reference plane. Following the SLOT calibration a single aluminium rod (5 mm diameter.) was placed in the tank to approximate a single pixel perturbation at a known location. Data was acquired with the rod in different positions in the tank and used to calibrate the propagation speed and attenuation of the coupling media. The matching solution (i.e., distilled water) in the 17 cm diameter Perspex container simulates specific propagation conditions.

**Fig. 14:**
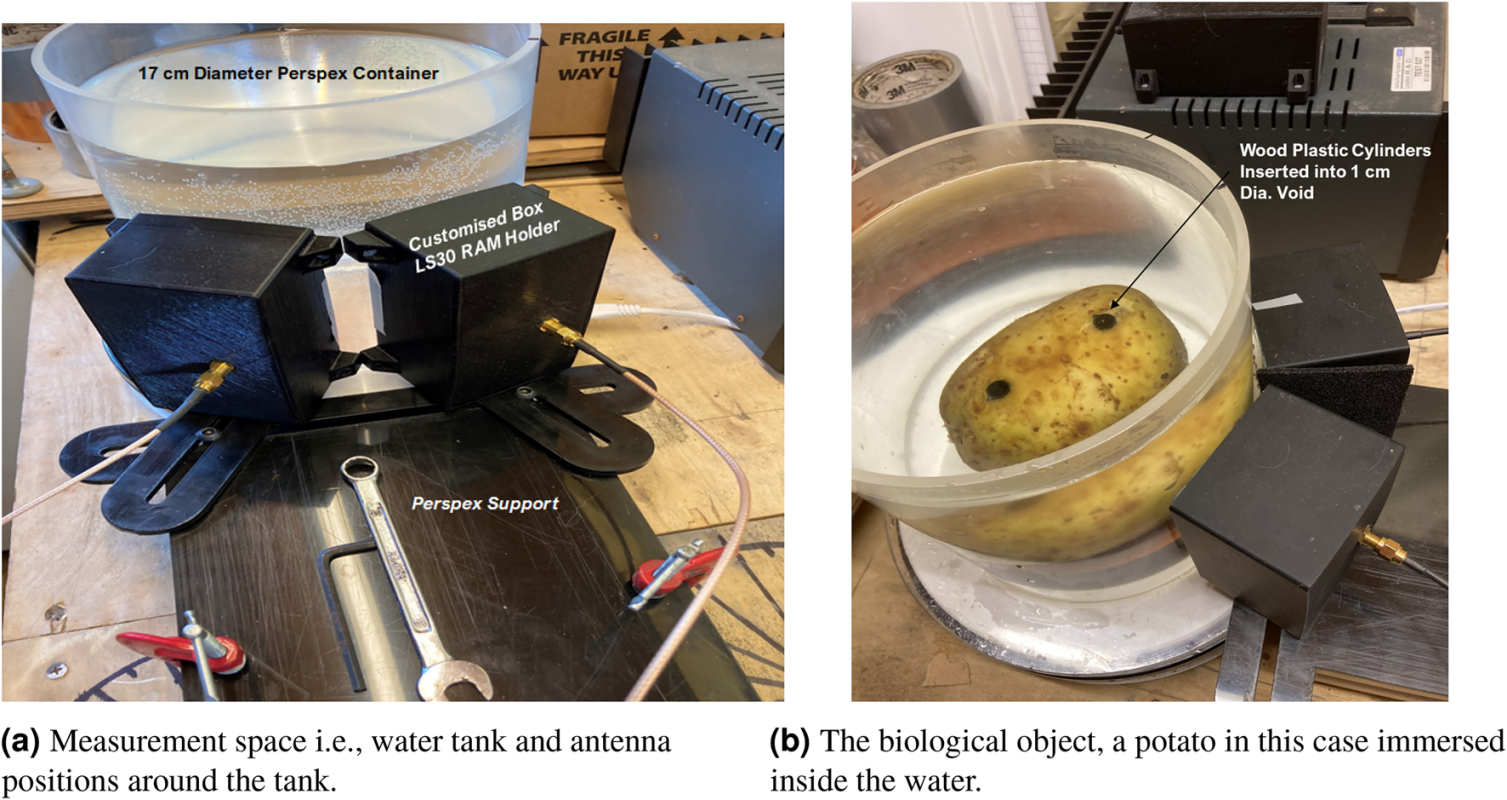
Real experimental setup depicting the measurement space, physical positions of the antennas, and placement of the object inside the matching liquid.

Figure 14a, b illustrate the real experimental setup detailing the components, antenna positions, the container of the matching liquid (water), and the arrangement of the target(s). This setup is crucial for conducting measurements using Inverse Synthetic Aperture Radar (ISAR) imaging. The biological elements chosen for this experiment are a potato and a turnip, selected to assess the potential of the proposed research. It is acknowledged that potatoes and turnips do not mimic the full anatomical and dielectric complexity of brain tissues. However, these biological surrogates were selected for their water content and structural heterogeneity, offering a practical testbed for spatial resolution. For instance, the relative permittivity ($\varepsilon _r$) of gray and white matter typically ranges from 40 to 60 depending on frequency, while water exhibits $\varepsilon _r \approx 80$, wood $\varepsilon _r \approx 2-5$, and metals approach infinity. These discrepancies affect the contrast mechanisms, backscatter intensity, and frequency dispersion characteristics. Thus, while valuable for evaluating spatial resolution and anomaly detectability, these models do not validate stroke detection capability. Therefore, future studies will incorporate multilayer agar or gelatin phantoms with frequency-dependent dielectric models of brain tissues. In the realm of microwave and electromagnetic theory, understanding how these biological elements, which contain significant water content, respond to measurements is essential. The long-term goal of this research is to apply the findings to measure real human heads for detecting strokes, where the head and strokes (blood clots) are different forms of water. The choice of biological elements aids in improving the algorithm and exploring diverse directions in this research endeavour. The measurements of the potato and turnip involve utilizing both forward and inverse models, enabling the recreation of two-dimensional microwave ISAR images of these biological samples. This foundational step with simpler biological elements paves the way for future advancements in human head imaging for medical applications.

### Results obtained from measured forward & inverse model

**Fig. 15:**
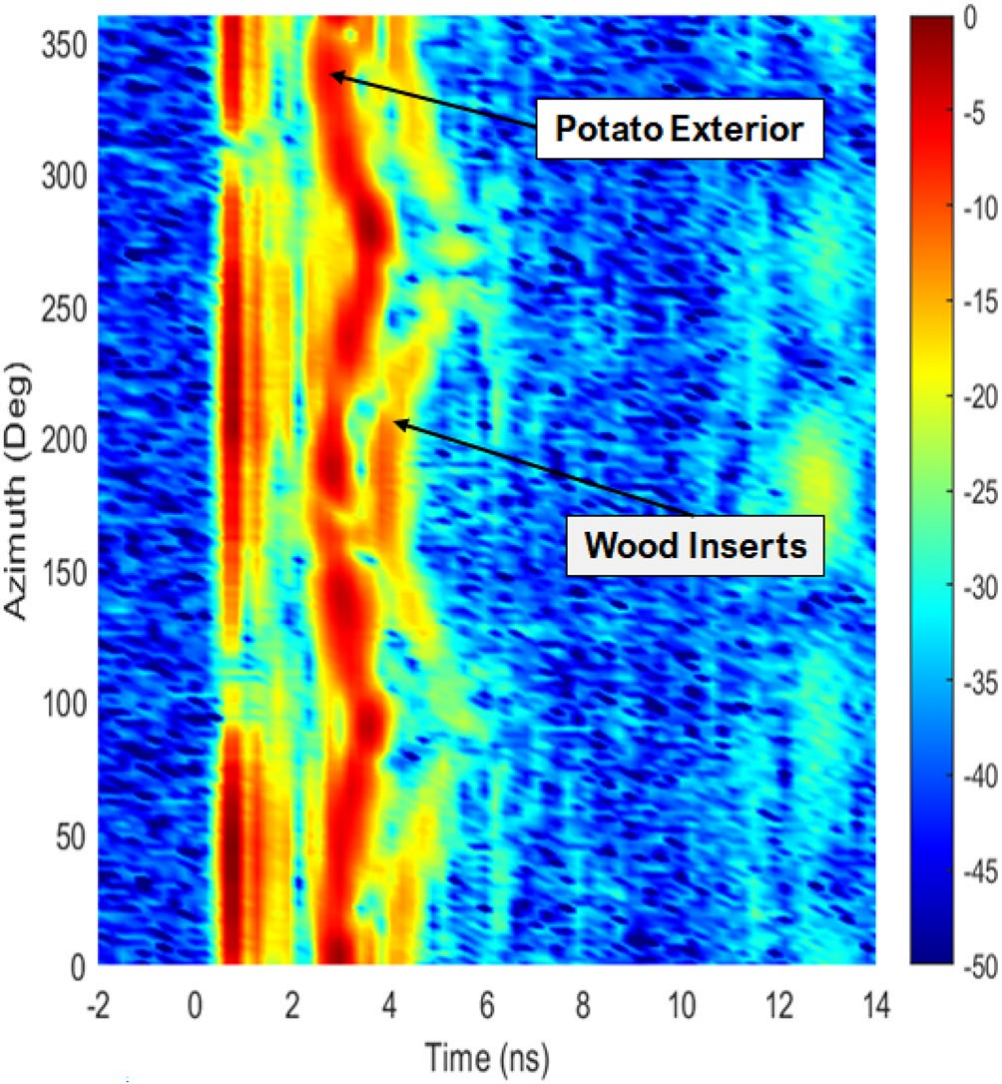
Forward model when a potato with two wood plastics insert measured.

Figure 15 illustrates the output of the forward model for a potato with physical dimensions of (8 cm $\times$ 12 cm), incorporating two wood inserts measuring 4 cm in length and 1 cm in diameter. The *x*-axis and *y*-axis represent the propagation delay (in nanoseconds) and azimuth angle of transmitting-receiving antenna positions (in degrees), respectively. The signals, sampled between 0.1 GHz to 4.8 GHz in a water medium with a relative permittivity of 80 (the same settings used for testing the algorithm for simulated environment in “Results obtained from simulated forward & inverse model”), take 14 ns (*x*-axis) to traverse the 17 cm diameter of the Perspex container. Therefore, the theoretical range resolution of the system $(\Delta R)$ is given by $\Delta R = v/2B$, where $v = c_0/\sqrt{\varepsilon _r}$. For air ($\varepsilon _r \approx 1$), $\Delta R \approx 31$ mm with B = 4.8 GHz, and for water ($\varepsilon _r \approx 80)$, $\Delta R \approx 3.5$ mm due to the lower propagation speed. This value may be slightly increased by the applied windowing to suppress sidelobes. The cross-range resolution of the system depends on aperture span and wavelength: for a $180^{\circ }$ synthetic aperture and central frequency 2.5 GHz, $\Delta x \approx 1.2$ cm. The sinograms in dB scale highlight three contrastable attenuators: the container wall itself, the exterior and interior of the potato, and the walls of the wood plastic cylinders, each appropriately labelled. Notably, the internal parts of the potato exhibit lower amplitudes compared to the three mentioned elements.

**Fig. 16:**
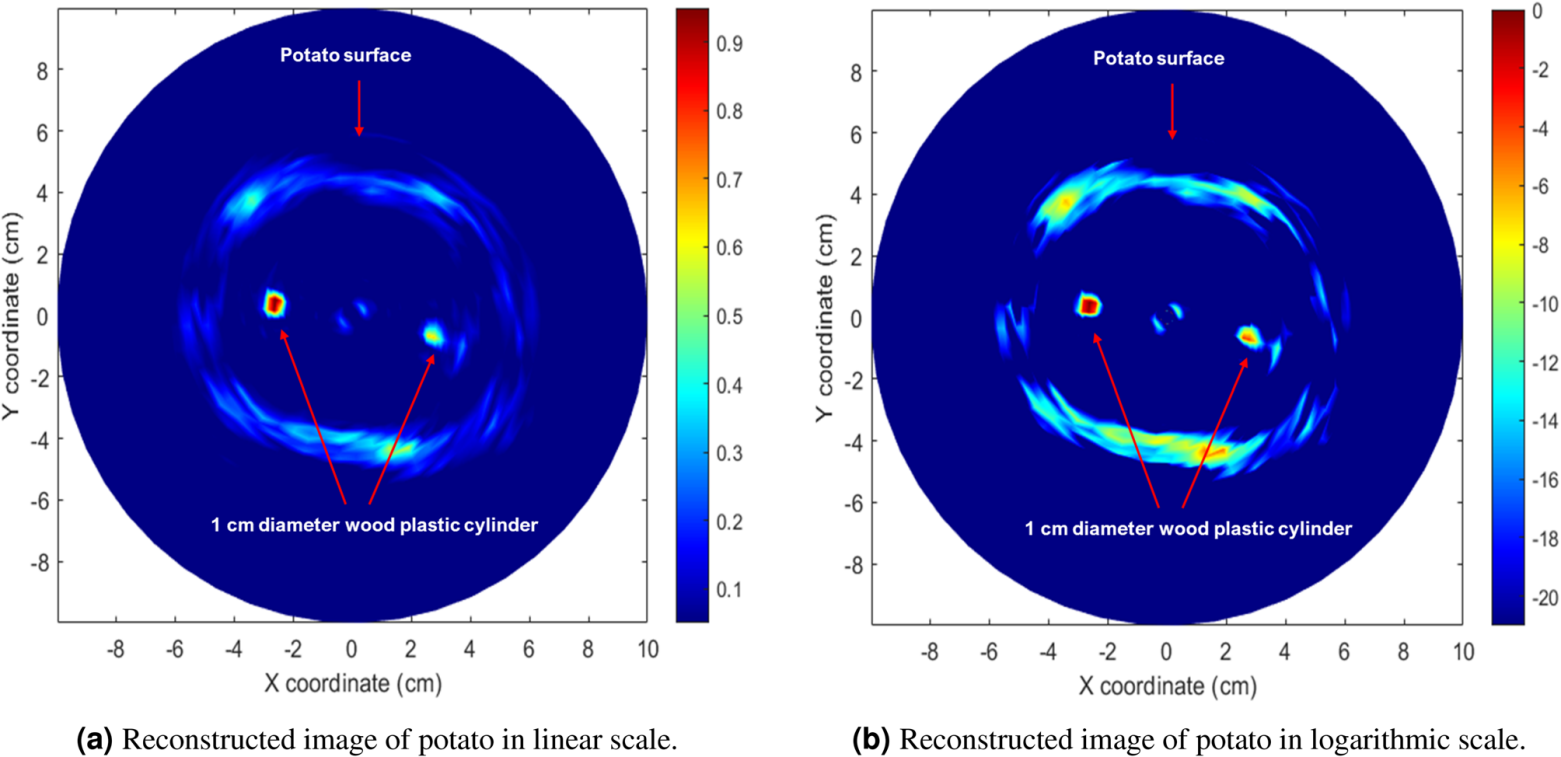
Reconstructed microwave ISAR image of the measured potato with two wood plastic inserts using the proposed method.

The reconstructed image of the potato, generated through the inverse model and back-projection of magnitudes in both linear and logarithmic scales, is presented in Fig. 16a, b. This visual depiction showcases a potato containing two wood plastic inserts with a diameter of 1cm each. The image’s brightness and contrast provide insights into the dielectric properties of both the potato and the wood plastic inserts, revealing how these elements respond to the electromagnetic field. The reconstructed potato image exhibits four distinct segments: the potato surface or skin, the two inserts, and the pith or inner medulla (bright section in the middle). Notably, other internal parts are not visible due to their interactions with the current experimental electromagnetic waves. The skin and pith act as a strong reflector, exhibiting relatively high contrast in dielectric properties compared to surrounding tissues like starch, nutrients, and water. Similarly, the two inserts serve as strong reflectors or attenuators. This suggests that abnormalities or diseases within the potato, exhibiting distinct dielectric properties from its surroundings, can be identified to some extent using microwave ISAR imaging techniques.

**Fig. 17:**
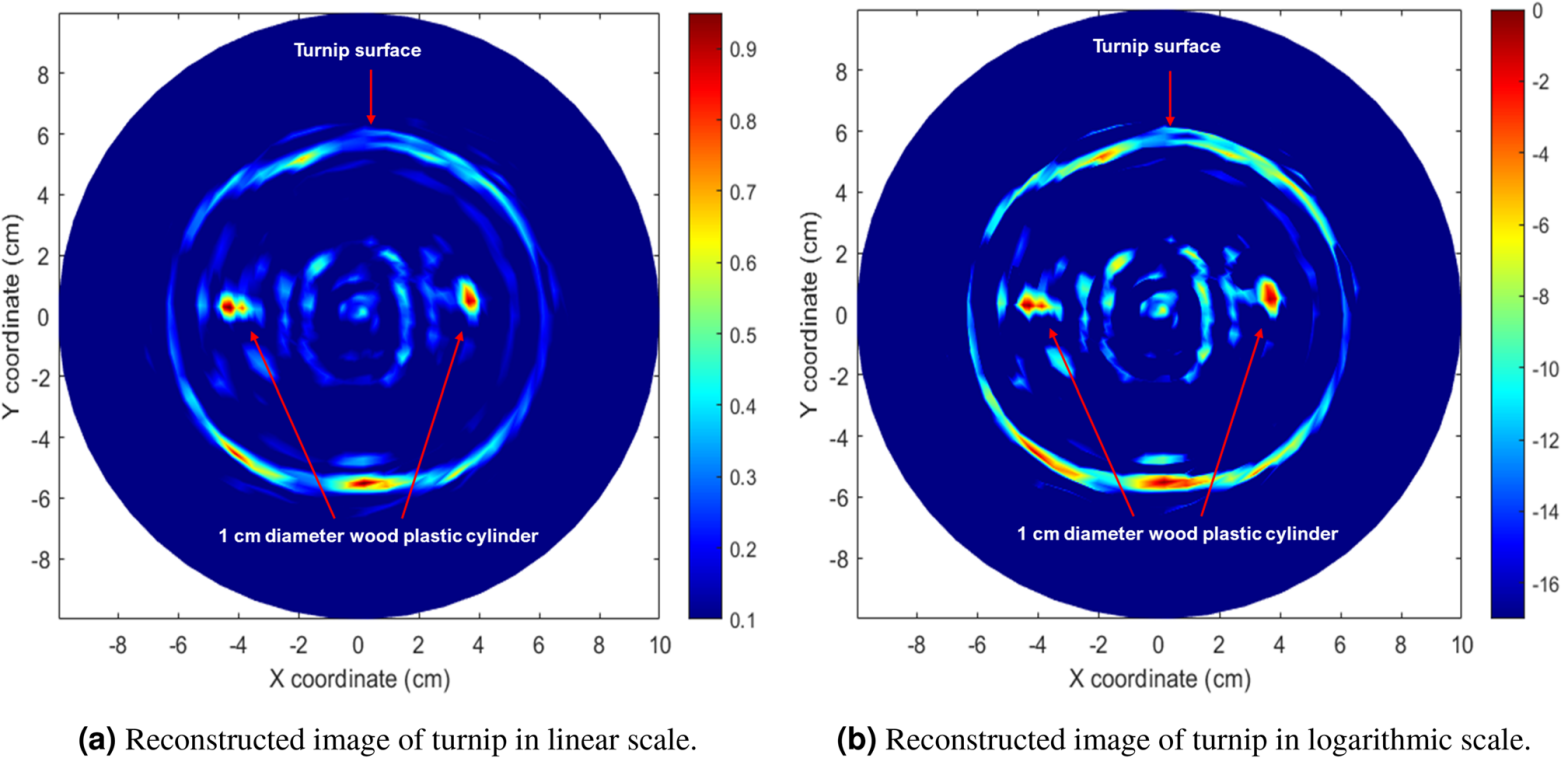
Reconstructed microwave ISAR image of the measured turnip with two wood plastic inserts using the proposed method.

The reconstructed microwave image of the same two wood plastics inserted into a turnip is depicted in Fig. 17a, b. Notably, more internal parts are discernible compared to the potato image. The turnip’s skin, sections of radially arranged vascular bundles and medullary rays, along with the two inserts, are identified through the proposed microwave imaging model. This suggests that the proposed imaging model has the capability to reveal details that can help identify normality and abnormality in turnips.

## Discussion & conclusion

The proposed research presents a pioneering endeavour towards developing a microwave ISAR imaging technique tailored for biological objects. The overarching objective is to expand this methodology for non-invasive and non-destructive human brain imaging, particularly for stroke detection. A specialized antenna has been crafted, utilising efficient design principles and incorporating RAM to minimize self-generated clutter. Water, selected for its ubiquitous availability and favourable permittivity-frequency relationship, serves as the matching liquid during dielectric property measurements. Its low cost and simplicity of use make it an ideal choice for coupling in experimental setups. While the proposed matrix inverse approach does not constitute a full non-linear dielectric reconstruction, it provides a computationally efficient and interpretable back-projection framework that employs a Born type approximation. A pseudo-inverse based method was used to reconstruct spatial scatterer maps, enabling rapid anomaly localization within 60 seconds. Although not algorithmically novel, this approach offers a practical alternative to computationally intensive iterative DBIM methods and is particularly well suited for early stage validation of the imaging system. This method offers significantly faster computation compared to conventional Newton-based methods, enhancing stroke detection and diagnosis potential. Moreover, the proposed model can be optimized for portability, facilitating deployment in ambulances for real-time imaging. The research methodology involves rigorous testing of forward and inverse models in simulated environments to gauge accuracy and capabilities. Subsequently, these models are applied to real biological objects such as potatoes and turnips to assess their efficacy in discerning internal structures. Preliminary results on biological surrogates demonstrate the system’s potential for resolving dielectric anomalies under controlled conditions, supporting its continued development toward clinical stroke detection.

Future research efforts will focus on advancing microwave ISAR imaging towards human brain stroke detection, while acknowledging the anatomical and electromagnetic complexity of cerebral tissue. In early-stage experiments, artificial inclusions such as wooden or plastic inserts were embedded in vegetable surrogates to evaluate spatial resolution and basic anomaly detectability. However, these models do not replicate the permittivity, conductivity, or scattering behaviour of real brain tissues. For instance, relative permittivity ($\varepsilon _r$) in brain tissues typically ranges from 40 to 60, while surrogate materials such as wood (dry wood $\varepsilon _r \approx 2$–5, wet wood $\varepsilon _r \approx 10$–30), water ($\varepsilon _r \approx 80$), and metals ($\infty$) differ significantly^32^. To improve biological realism, future studies will involve multilayer agar or gelatin based phantoms that mimic tissue specific dielectric dispersion and anatomical structure. These phantoms will incorporate regions emulating healthy and pathological brain matter to better assess contrast resolution and imaging fidelity in stroke-like conditions. Additionally, biological objects with high water content and layered internal structure such as watermelon, cabbage, and onion will be used to validate system performance under diverse scattering environments. To enhance measurement realism, investigations will be extended to explore alternative coupling media such as glycerol and saline water, which provide better impedance matching and reduced signal attenuation compared to pure water. Pulse rectification techniques will also be integrated to improve signal-to-noise ratio and image clarity. Efforts will be directed toward constructing sensitivity matrices based on the dielectric properties of real, heterogeneous objects, rather than idealized or simulated ones, thereby better modelling measurement uncertainty and system noise. The current pseudo-inverse solution using SVD will be compared with other regularization techniques, including the least squares and penalized least squares methods, to enhance robustness and minimize inversion artefacts. Collectively, these future directions aim to transition the imaging system from proof-of-concept to practical application, ultimately enabling biologically accurate, contrast-sensitive microwave ISAR imaging for human brain diagnostics and stroke localization.

## Data Availability

The datasets used and/or analysed during the current study available from the corresponding author on reasonable request.
